# An evaluation of the Zambia influenza sentinel surveillance system, 2011–2017

**DOI:** 10.1186/s12913-019-4884-5

**Published:** 2020-01-13

**Authors:** Paul Simusika, Stefano Tempia, Edward Chentulo, Lauren Polansky, Mazyanga Lucy Mazaba, Idah Ndumba, Quinn K. Mbewe, Mwaka Monze

**Affiliations:** 1 National Influenza Center, Virology Laboratory, University Teaching Hospital, Lusaka, Zambia; 2 Influenza Division, Centers for Disease Control and Prevention, Atlanta, GA USA; 3 Influenza Program, Centers for Disease Control and Prevention, Pretoria, South Africa; 4 MassGenics, Duluth, GA USA

**Keywords:** Influenza, Surveillance, Evaluation, Zambia

## Abstract

**Background:**

Over the past decade, influenza surveillance has been established in several African countries including Zambia. However, information on the on data quality and reliability of established influenza surveillance systems in Africa are limited. Such information would enable countries to assess the performance of their surveillance systems, identify shortfalls for improvement and provide evidence of data reliability for policy making and public health interventions.

**Methods:**

We used the Centers for Disease Control and Prevention guidelines to evaluate the performance of the influenza surveillance system (ISS) in Zambia during 2011–2017 using 9 attributes: (i) data quality and completeness, (ii) timeliness, (iii) representativeness, (iv) flexibility, (v) simplicity, (vi) acceptability, (vii) stability, (viii) utility, and (ix) sustainability. Each attribute was evaluated using pre-defined indicators. For each indicator we obtained the proportion (expressed as percentage) of the outcome of interest over the total. A scale from 1 to 3 was used to provide a score for each attribute as follows: < 60% (as obtained in the calculation above) scored 1 (weak performance); 60–79% scored 2 (moderate performance); ≥80% scored 3 (good performance). An overall score for each attribute and the ISS was obtained by averaging the scores of all evaluated attributes.

**Results:**

The overall mean score for the ISS in Zambia was 2.6. Key strengths of the system were the quality of data generated (score: 2.9), its flexibility (score: 3.0) especially to monitor viral pathogens other than influenza viruses, its simplicity (score: 2.8), acceptability (score: 3.0) and stability (score: 2.6) over the review period and its relatively low cost ($310,000 per annum). Identified weaknesses related mainly to geographic representativeness (score: 2.0), timeliness (score: 2.5), especially in shipment of samples from remote sites, and sustainability (score: 1.0) in the absence of external funds.

**Conclusions:**

The system performed moderately well in our evaluation. Key improvements would include improvements in the timeliness of samples shipments and geographical coverage. However, these improvements would result in increased cost and logistical complexity. The ISSS in Zambia is largely reliant on external funds and the acceptability of maintaining the surveillance system through national funds would require evaluation.

## Background

Seasonal influenza virus infections are responsible for an estimated 291,243–645,832 respiratory deaths globally every year [1]. Influenza infections have been described in Zambia [2] since the 1990s, but because of limited laboratory diagnostic capacity prior to 2008 [3], little was known about the contribution of influenza viruses to the respiratory disease burden, rendering difficult public health planning for the prevention and control of influenza-associated illness. At present, no influenza treatment or immunization guidelines are available in Zambia, and no measures for mitigating the transmission and disease burden associated with influenza infection are in place.

Public health surveillance is the ongoing, systematic collection, analysis, interpretation, and dissemination of data regarding a health-related event for use in public health action to reduce morbidity and mortality and to improve health [4]. Data disseminated by a public health surveillance system can be used for immediate public health action, program planning and evaluation, and formulation of research hypotheses [5].

The lack of a routine influenza surveillance program in Zambia placed the country at a disadvantage as such a system would serve as an ever-ready early warning system with the potential to detect and confirm the etiology of respiratory disease outbreaks, including seasonal and pandemic influenza viruses, and trigger a response. The Ministry of Health (MoH) recognized a need to gather influenza epidemiological and virological data from Zambia that would not only help decision makers to formulate policies targeted toward influenza prevention in Zambia but also to provide a more comprehensive understanding of the dynamics of influenza viruses worldwide and in African tropical zones in particular. In addition, virological data would help to identify seed viruses for the production of seasonal influenza vaccines that can be effectively used for the Southern Hemisphere. In recognition of these needs, in 2008 the Zambia Ministry of Health established an influenza sentinel surveillance system (ISSS) targeting outpatients with influenza-like illness (ILI) and in-patients with severe acute respiratory illness (SARI).

The World Health Organization (WHO) recommends that established influenza surveillance systems undergo a comprehensive evaluation periodically, beginning 1–2 years after implementation in order to ascertain how well the system fulfills its purposes [6, 7]. Such evaluations are useful to identify shortfalls, improve performance and provide evidence of data reliability for policy making and public health interventions. However, despite recent progress in describing the epidemiology and burden of influenza in sub-Saharan Africa [8–16], only a few countries have implemented a comprehensive evaluation of their surveillance systems [17–21].

To ensure that the national ISSS objectives are met, that they are in line with WHO requirements and that reliable data are generated for public health interventions, we conducted an evaluation of the performance of the Zambia ILI and SARI ISSS from January 2011 to December 2017. The inception and consolidation of the surveillance system occurred during 2008–2010. The outputs of the surveillance systems during this period have been previously published [3] and they are not included in this review.

## Methods

### Overview of the influenza sentinel surveillance system during 2011–2017

**Fig. 1:**
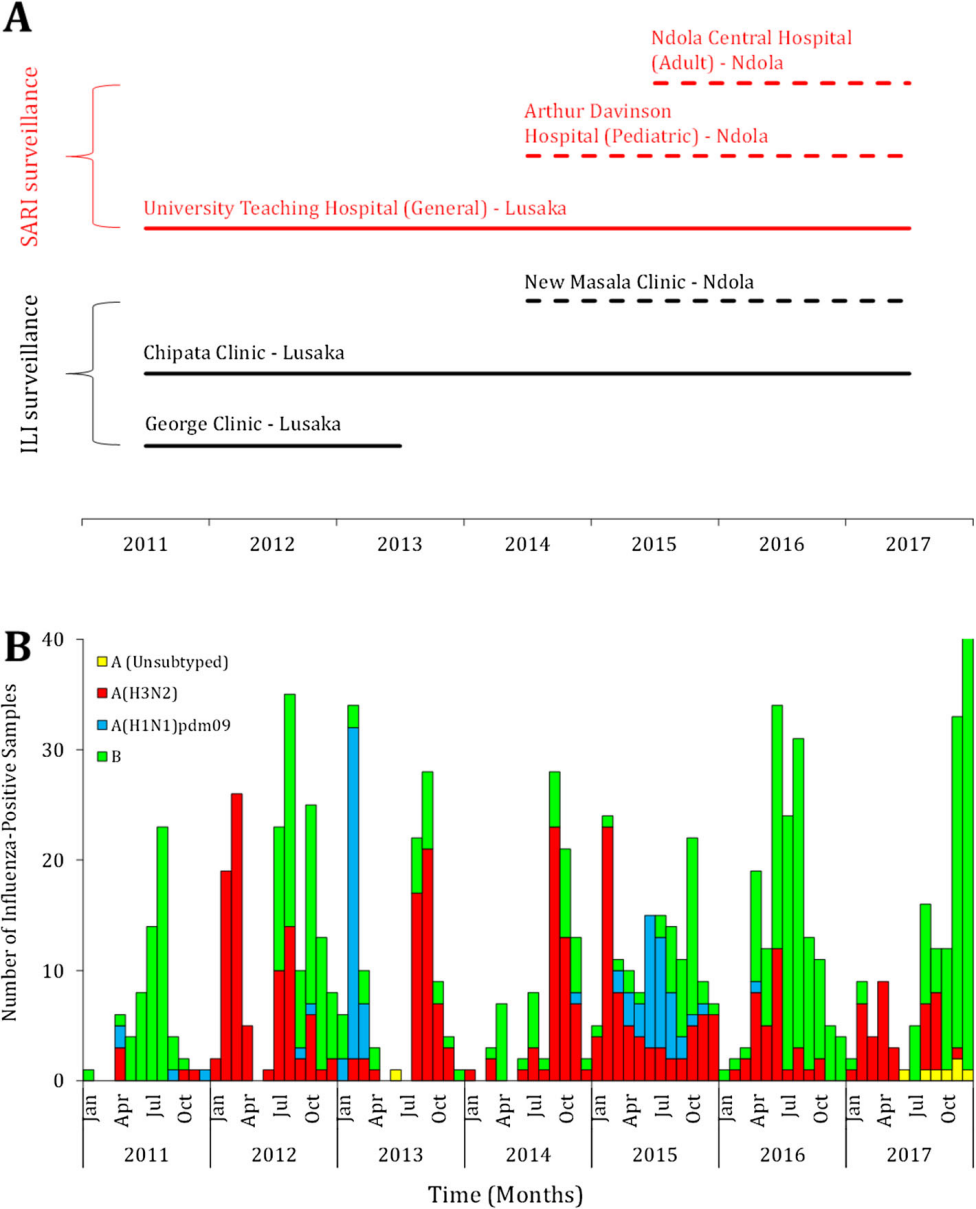
Influenza sentinel surveillance implemented at six surveillance sites in Zambia, 2011–2017. **A:** years of implementation of influenza surveillance by sentinel site (solid line: sentinel sites situated in Lusaka Province; dashed line: sentinel sites situated in Copperbelt Province). **B:** monthly number of influenza-positive specimens among patients with influenza-like illness (ILI) or severe acute respiratory illness (SARI)

In Zambia, an ISSS was first established in 2008 in Lusaka Province (the most populated province of the country where the capital city is located) [3] and subsequently expanded to the Copperbelt Province (the second most populated province). The objectives of the Zambia-ISSS were to: (i) monitor the temporal trends of influenza virus circulation; (ii) monitor the circulating influenza virus types and subtypes annually, including pandemic strains; (iii) assess the proportion of patients meeting the ILI and SARI case definition attributable to influenza virus infection; (iv) assess risk factors for influenza-associated severe illness; (v) assess the burden of influenza-associated illness; and (vi) obtain and share clinical samples for annual selection of influenza virus strains for influenza vaccine formulation under the WHO-Global Influenza Surveillance and Response Network. During 2011–2017, ILI influenza sentinel surveillance was conducted in three urban outpatient clinics targeting outpatients with ILI and three large referral hospitals targeting inpatients with SARI (Fig. 1, panel A). Both pediatric and adult cases were investigated.

A case of ILI was defined as an outpatient of any age presenting with a recorded temperature ≥ 38 °C and cough or sore throat of duration of ≤7 days [6]. A case of SARI was defined as a hospitalized person who had illness onset within 7 days of admission and who met age-specific clinical inclusion criteria. A case in children aged 2 days to < 5 years included any hospitalized patient with cough or difficulty breathing and at least one of the following danger signs: unable to drink or breastfeed, lethargic, vomits everything, convulsion, chest in-drawing or stridor in a calm child. A case in persons aged ≥5 years included any hospitalized patient with fever (≥38 °C), cough and shortness of breath or difficulty breathing [6]. All patients with SARI were eligible for enrollment; whereas 5 patients with ILI (the first patient every day from Monday to Friday) were targeted for enrollment per week and per facility.

Activities at the sentinel sites situated in Lusaka were coordinated by a dedicated surveillance officer from the National Influenza Center (NIC). For sites situated in the Copperbelt Province, supervision was conducted by a focal point at the Provincial Medical Office. Due to high staff turnover, training was conducted once a year while supervisory visits to sites were done at least quarterly. Clinical staff at sentinel sites tasked with surveillance implementation were remunerated according to time spent on surveillance activities. The Zambia ISSS is co-funded by the Zambia-MoH, the U.S. Centers for Disease Control and Prevention (U.S. CDC) and WHO.

#### Data and sample collection procedures

Data were collected from eligible cases using WHO adapted and standardized case investigation forms (CIF) by trained nurses in each sentinel site. For patients meeting the SARI case definition the number of patients eligible for enrollment was also collected. A combination of nasopharyngeal and oropharyngeal swabs were collected from consenting patients, placed in universal transport medium (Copan, Murrieta, California, USA) and transported to the University Teaching Hospital Virology Laboratory, the WHO-recognized NIC in Lusaka, via a cold chain for testing. Samples with respective CIF forms collected from sites situated in Lusaka were sent to the laboratory within 48 h, while those from the Copperbelt Province were refrigerated and sent within two weeks of collection [22]. Verbal informed consent was obtained from all patients prior to data and specimen collection. For children aged < 15 years, verbal consent was obtained from a parent or legal guardian.

#### Sample processing

At the NIC, samples and accompanying CIFs were logged by recording key data in the laboratory log book. Specimens were tested for the presence of influenza viruses using the U.S. CDC real-time reverse-transcription polymerase chain reaction (RT-PCR) protocol for characterization of influenza viruses [3]. During 2011–2012, all specimens from children aged < 5 years with SARI were tested for other respiratory viruses including parainfluenza virus (PIV) types 1, 2 and 3, respiratory syncytial virus (RSV), adenovirus, rhinovirus, human metapneumovirus (hMPV), coronavirus (OC43, NL63, 229E, HKU1) and bocavirus using an FTD multiplex real-time RT-PCR assay [23]. Since 2012, RT-PCR influenza-positive samples with a cycle threshold value < 28 were cultured at the NIC, after which further viral identification of the samples was done using Haemagglutination (HA) and Haemagglutination Inhibition (HAI) testing. Confirmed virus isolates were shared with WHO Collaborating Centers for further characterization; both viral isolates and clinical specimens were shared twice a year.

#### Data management and reporting

Data from the CIF including demographics and clinical information, exposure risk and past medical history were captured in a customized influenza sentinel surveillance MS Access® database with inbuilt data validation checks. Data entry was done daily as forms were received. Monthly reports of the total number of samples received and processed (including positive specimens by subtype) were reported to the Ministry of Health public health units. A separate patient line-list was also sent monthly to the sentinel sites; the results report included total samples processed by sentinel site and total confirmed influenza infections. Weekly influenza surveillance data and influenza-positive specimens were reported to WHO AFRO using an excel template. Virological and epidemiological data were reported to WHO FLUNET / FLUID.

### Evaluation of the influenza sentinel surveillance system

The evaluation of the ISSS was based on CDC guidelines [5] and focused on the performance of the system from January 2011 to December 2017. The objective of the evaluation was to determine: (i) whether the surveillance system was designed and operated in such a way as to be capable of detecting and monitoring seasonal influenza viruses, including pandemic strains; (ii) the usefulness of the collected data to assess influenza disease burden in the general population; (iii) the impact of the system in informing public health intervention and policies; and (iv) the ability of the system to contribute to the annual selection of influenza strains for vaccine development. The performance of the system was assessed using nine surveillance attributes, namely: (i) data quality and completeness for selected key variables, (ii) timeliness, (iii) representativeness, (iv) flexibility, (v) simplicity (vi) acceptability, (vii) stability; (viii) utility; and (ix) sustainability.

For consistency and comparability of findings we used the evaluation method and scoring system utilized for influenza surveillance evaluations conducted in other African countries [17–21]. Each of the above mentioned attribute was evaluated using pre-defined quantitative or qualitative indicators. A total of 38 indicators were developed for the evaluation. The number of indicators evaluated for each attribute is provided in Table 1; whereas the individual indicators and the calculations and data sources used to evaluate each indicator are provided in Tables 2-5.

**Table 1:** Mean indicators’ scores (range 1–3) for each attribute used for the evaluation of the influenza sentinel surveillance system in Zambia, 2011-2017^a^

Attributes	Number of evaluated indicators	Mean score	Performance
• Data quality and completeness	7	2.9	Moderate to good
• Timeliness	2	2.5	Moderate
• Representativeness	2	2.0	Moderate to weak
• Flexibility	2	3.0	Good
• Simplicity	7	2.8	Moderate to good
• Acceptability	4	3.0	Good
• Stability	8	2.6	Moderate to good
• Utility	4	2.7	Moderate to good
• Sustainability	2	1.0	Weak
• Overall	38	2.6	Moderate to good

^a^Each quantitative indicator was evaluated as the proportion (expressed as percentage) of the outcome of interest over the total. A scale from 1 to 3 was used to provide a score for each quantitative indicator as follows: < 60% (from the above calculation) scored 1 (weak performance); 60–79% scored 2 (moderate performance); ≥80% scored 3 (good performance). For indicators for which a proportion over a total could not be obtained (qualitative indicators) a score was assigned based on the same scale using expert consensus. The scores assigned to each indicator were averaged for all indicators evaluated for each attribute to provide a mean score for each surveillance attribute. An overall score for the surveillance system was obtained by averaging the scores of all evaluated indicators

**Table 2:** Indicators for data quality and completeness, timeliness, representativeness and flexibility used for the evaluation of the influenza sentinel surveillance system in Zambia, 2011-2017^a^

Indicator	Calculation/data inputs	Data source	Indicator value	Score
Data quality and completeness				
• Proportion of enrolled patients with ILI against set target (5 patients per week per site).	Number of enrolled patients with ILI / Estimated target.	ISS database and estimated target	140.4%	3
• Proportion of enrolled patients with SARI against set target (all eligible patients).	Number of enrolled patients with SARI / All patients with SARI.	ISS database	69.8%	2
• Proportion of SARI/ILI cases that meet the case definition	Number of ILI/SARI cases that meet the case definition / Total number of enrolled ILI/SARI cases	ISS database	86.5%	3
• Proportion of forms without at least one inconsistent or missing value for key variables^b^	Number of forms without at least one incorrect or missing value / Total number of forms	ISS database	93.8%	3
• Proportion of sampled ILI/SARI cases with available laboratory results	Number of ILI/SARI cases with available laboratory results / Number of sampled ILI/SARI cases	ISS database	93.2%	3
• Proportion of sample with positive RNP results	Number of samples with a positive RNP result / Total number of samples tested	ISS database	99.8%	3
• Proportion of collected variables included in the WHO recommended minimum data collection standard	Number of collected variables within the WHO list / Number of WHO recommended variables.	CIF and WHO guidelines for influenza sentinel surveillance.	82.3%	3
Timeliness				
• Proportion of samples received within the target period from collection^c^	Number of samples received at the laboratory within 14 days from collection / Number of samples received	ISS database	73.2%	2
• Proportion of samples tested within one week from receipt	Number of samples tested within two weeks from receipt / Number of samples tested	ISS database	87.3%	3
Representativeness				
• Geographical coverage	Number of provinces covered by the influenza sentinel surveillance network / Total number of provinces of the country	Geographic distribution of sentinel sites.	20.0%	1
• Inclusion of all age groups	Age distribution of ILI/SARI cases (median, minimum and maximum)	ISS database	Med.: 4 Y	3
			Min.: 0 Y	
			Max.: 97 Y	
Flexibility				
• Expansion of sentinel sites participating to the ISSS since inception	Number of new sentinel sites since inception	Protocol	5	3
• Surveillance for pathogens other than influenza	Number of investigated pathogens other than influenza	Protocol	10	3

Abbreviations: *ILI* influenza-like-illness; *SARI* severe acute respiratory illness; *CIF* case investigation form; *RNP* RiboNucleic Protein; *WHO* World Health Organization; *ISS* influenza sentinel surveillance

^a^Each quantitative indicator was evaluated as the proportion (expressed as percentage) of the outcome of interest over the total (indicator value). A scale from 1 to 3 was used to provide a score for each quantitative indicator as follows: < 60% (from the above calculation) scored 1 (weak performance); 60–79% scored 2 (moderate performance); ≥80% scored 3 (good performance)

^b^Key variables evaluated for completeness and consistency of data: site, age/date of birth, sex, date of consultation/admission, date of symptoms onset, date of sample collection and signs and symptoms included in the case definitions

^c^The target period was 48 h from sites situated in Lusaka Province and 14 days from sites situated in Copperbelt Province

**Table 3:** Indicators for simplicity and acceptability used for the evaluation of the influenza sentinel surveillance system in Zambia, 2011-2017^a^

Indicator	Calculation/data inputs	Data source	Indicator value	Score
Simplicity				
• Perception of surveillance staff on identification of cases	Number of surveillance staff within each reported category [very difficult (VD), difficult (D), easy (E), very easy (VE)] / Number of surveillance staff interviewed	Questionnaire survey among surveillance staff at sentinel sites	VD: 9.3%	3
			D: 7.0%	
			E: 27.9%	
			VE: 55.8%	
• Perception of surveillance staff on obtaining consent	Number of surveillance staff within each reported category [very difficult (VD), difficult (D), easy (E), very easy (VE)] / Number of surveillance staff interviewed	Questionnaire survey among surveillance staff at sentinel sites	VD: 4.6%	3
			D: 13.3%	
			E: 35.6%	
			VE: 46.5%	
• Perception of surveillance staff on completing the CIF	Number of surveillance staff within each reported category [very difficult (VD), difficult (D), easy (E), very easy (VE)] / Number of surveillance staff interviewed	Questionnaire survey among surveillance staff at sentinel sites	VD: 0.0%	3
			D: 0.0%	
			E: 27.9%	
			VD: 72.1%	
• Perception of surveillance staff on sample collection	Number of surveillance staff within each reported category [very difficult (VD), difficult (D), easy (E), very easy (VE)] / Number of surveillance staff interviewed	Questionnaire survey among surveillance staff at sentinel sites	VD: 0.0%	3
			D: 5.7%	
			E: 80.0%	
			VE: 14.3%	
• Perception of surveillance staff on packaging and storage of samples	Number of surveillance staff within each reported category [very difficult (VD), difficult (D), easy (E), very easy (VE)] / Number of surveillance staff interviewed	Questionnaire survey among surveillance staff at sentinel sites	VD: 0.0%	3
			D: 0.0%	
			E: 20.9%	
			VE: 79.1%	
• Perception of surveillance staff on completing the screening/enrollment logbook	Number of surveillance staff within each reported category [very difficult (VD), difficult (D), easy (E), very easy (VE)] / Number of surveillance staff interviewed	Questionnaire survey among surveillance staff at sentinel sites	VD: 0.0%	3
			D: 0.0%	
			E: 72.4%	
			VE: 27.6%	
• Time to enroll a SARI/ILI case from patient’s identification to the sample packaging	Number of surveillance staff within each reported category (< 30 min, 30–60 min, > 60 min) / Number of surveillance staff interviewed	Questionnaire for surveillance staff at sentinel sites	< 30: 68.6%	2
			30–60: 20.0%	
			> 60: 11.4%	
Acceptability				
• Proportion of surveillance staff that is satisfied with the weekly bulletins	Number of surveillance staff within each reported category [not satisfied (NS), poorly satisfied (PS), satisfied (S), very satisfied (VS)] / Number of surveillance staff interviewed	Questionnaire for surveillance staff at sentinel sites	NS: 0.0%	3
			PS: 0.0%	
			S: 25.6%	
			VS: 74.4%	
• Proportion of surveillance staff that is satisfied with the feedback of laboratory results	Number of surveillance staff within each reported category [not satisfied (NS), poorly satisfied (PS), satisfied (S), very satisfied (VS)] / Number of surveillance staff interviewed	Questionnaire for surveillance staff at sentinel sites	NS: 0.0%	3
			PS: 9.3%	
			S: 69.8%	
			VS: 20.9%	
• Proportion of time allocated to influenza surveillance activities per week	Number of hours allocated to influenza surveillance activities per week / Number of working hour per week	Questionnaire for surveillance staff at sentinel sites	22.5%	3
• Number of ILI/SARI patients enrolled per day	Number of surveillance staff within each reported category [≤5 patients (≤5), 6–10 patients (6–10), > 10 (> 10)] / Number of surveillance staff interviewed	Questionnaire for surveillance staff at sentinel sites	≤5: 95.2%	3
			6–10: 3.2%	
			> 10: 1.2%	

Abbreviations: *ILI* influenza-like-illness; *SARI* severe acute respiratory illness; *CIF* Case Investigation Form

^a^Each quantitative indicator was evaluated as the proportion (expressed as percentage) of the outcome of interest over the total (indicator value). A scale from 1 to 3 was used to provide a score for each quantitative indicator as follows: < 60% (from the above calculation) scored 1 (weak performance); 60–79% scored 2 (moderate performance); ≥80% scored 3 (good performance)

**Table 4:** Indicators for stability used for the evaluation of the influenza sentinel surveillance system in Zambia, 2011-2017^a^

Indicator	Calculation/data inputs	Data source	Indicator value	Score
Stability				
• Frequency of lack of data collection forms	Number of surveillance sites within each reported category [never (0), once per year (1), 2–3 times per year (2–3), ≥4 times per year(≥4)] / Number of surveillance sites	Questionnaire for surveillance staff at sentinel sites	0: 83.3%	3
			1: 16.7%	
			2–3: 0.0%	
			≥4: 0.0%	
• Frequency of lack of sampling material	Number of surveillance sites within each reported category [never (0), once per year (1), 2–3 times per year (2–3), ≥4 times per year(≥4)] / Number of surveillance sites	Questionnaire for surveillance staff at sentinel sites	0: 83.3%	3
			1: 16.7%	
			2–3: 0.0%	
			≥4: 0.0%	
• Frequency at which a power failure, including the generator, occurred at the surveillance sites	Number of surveillance sites within each reported category [never (N), seldom (S), often (O), regularly (R)] / Number of surveillance sites	Questionnaire for surveillance staff at sentinel sites	N: 16.7%	2
			S: 50.0%	
			O: 33.3%	
			R: 0.0%	
• Proportion of sentinel sites with at least one member of staff trained on sentinel surveillance procedures	Number of sentinel sites with at least one trained member of staff / Number of surveillance sites	Questionnaire for surveillance staff at sentinel sites	100.0%	3
• Proportion of sentinel surveillance staff ever trained on sentinel surveillance procedures	Number of surveillance staff ever trained / Number of surveillance staff	Questionnaire for surveillance staff at sentinel sites	90.7%	3
• Proportion of sentinel surveillance staff trained on sentinel surveillance procedures during the last one year	Number of surveillance staff during the last one year / Number of surveillance staff	Questionnaire for surveillance staff at sentinel sites	69.7%	2
• Availability and use of standard operating procedures (SOPs) by surveillance staff	Number of surveillance staff with access and use of SOPs / Number of surveillance staff	Questionnaire for surveillance staff at sentinel sites	97.7%	3
• Proportion of surveillance sites providing samples weekly after 3 months from inception	Number of surveillance sites providing samples weekly/ Number of surveillance sites	ISS database	100.0%	3
• Proportion of weekly surveillance reports sent to MoH	Number of surveillance reports sent to MoH / Number of reporting weeks	Surveillance reports	71.4%	2

Abbreviations: *ISS* influenza sentinel surveillance; *MoH* Ministry of Health

^a^Each quantitative indicator was evaluated as the proportion (expressed as percentage) of the outcome of interest over the total (indicator value). A scale from 1 to 3 was used to provide a score for each quantitative indicator as follows: < 60% (from the above calculation) scored 1 (weak performance); 60–79% scored 2 (moderate performance); ≥80% scored 3 (good performance)

**Table 5:** Indicators for utility and sustainability used for the evaluation of the influenza sentinel surveillance system in Zambia, 2011-2017^a^

Indicator	Calculation/data inputs	Data source	Indicator value	Score
Utility				
*International*				
• Proportion of weeks with data reported to WHO FluNet	Number of weeks with data reported to WHO FluNet / Number of weeks during the evaluated period	Weekly FluNet submissions	98.4%	3
• Mean annual number of samples shared with WHO Collaborating Centers (WHO CC)	Number of samples/isolates shared with WHO CC / Number of years with samples shipped.	Shipment logs to WHO CC London	23 (range 14–33) shipped during 2012 and 2014–2017	3
• Number of contributions to influenza Regional/Global studies	Number of publications on Regional/Global studies with influenza data from Zambia	PubMed	2 [8,28]	2
*Domestic*				
• Ability to assess important influenza epidemiological features/public health outcomes	• Temporal patters of influenza virus circulation (Yes) [3]	Publications and reports	80.0%^b^	3
	• Circulating influenza types/subtypes, including pandemic strains (Yes) [3]			
	• Proportion of ILI/SARI illness attributable to influenza virus infection (Yes) [3]			
	• Risk factors for influenza-associated severe illness (No)			
	• Burden of influenza-associated illness (Yes) [14]			
Sustainability				
• Proportion of the ISSS cost covered by the Zambia-MoH	Cost covered by the Zambia-MoH / Total cost	Budget report	16.9%	1
• Availability and implementation of a sustainability plan	• Drafted (Yes)	Sustainability plan	25.0%^b^	1
	• Finalized (No)			
	• Approved (No)			
	• Implemented (No)			

Abbreviations: *MoH* Ministry of Health

^a^Each quantitative indicator was evaluated as the proportion (expressed as percentage) of the outcome of interest over the total. A scale from 1 to 3 was used to provide a score for each quantitative indicator as follows: < 60% (from the above calculation) scored 1 (weak performance); 60–79% scored 2 (moderate performance); ≥80% scored 3 (good performance). For indicators for which a proportion over a total could not be obtained (qualitative indicators) a score was assigned based on the same scale using expert consensus

^b^Indicator value calculated by dividing the number of achieved outcome by the total number of outcome considered (i.e. 4/5 = 80.0% or 1/4 = 25.0%)

Data for calculation of the indicators for data quality and completeness, timeliness, stability and utility were obtained from various sources including the main influenza sentinel surveillance database, the laboratory database, annual reports and other documents and records. In order to assess simplicity, acceptability, stability and utility, a self-administered questionnaire as shown in additional file 1 below was designed targeting staff involved in surveillance at the sentinel sites. The questionnaire was designed to capture data based on staff perceptions of the program. Data collected from the surveillance system were also compared with WHO minimum data collection standards for ILI and SARI surveillance [6].

For each quantitative indicator we first obtained the proportion (expressed as percentage) of the outcome of interest over the total [18–21]. For instance, for the indicator on completeness of laboratory testing (one of the indicators used to evaluate the data quality and completeness attribute) we divided the number of samples with available influenza results by the total number of samples collected and received by the laboratory. Subsequently, similar to other influenza surveillance evaluations conducted in Africa, we used a scale from 1 to 3 to provide a score for each quantitative indicator as follows: < 60% (as obtained in the example above) scored 1 (weak performance); 60–79% scored 2 (moderate performance); ≥80% scored 3 (good performance) [18–21]. For indicators for which a proportion over a total could not be obtained (qualitative indicators) a score was assigned based on the same scale using expert consensus.

Thereafter, the scores assigned to each indicator were averaged for all indicators evaluated within each attribute to provide an overall score for each surveillance attribute assessed in this study. An overall score for the surveillance system was obtained by averaging the scores of all evaluated indicators as previously described [18–21]. All data generated by the surveillance system during the review period were included in the evaluation. The analysis was implemented using Stata version 14.2 (StataCorp, College Station, Texas, USA).

### Ethical approval and consent to participate

This surveillance evaluation was deemed non-research by the Zambia-MoH and the US CDC. Consent to participate was voluntary and verbally obtained, because this was a non-research and the procedure was non-risks to client ethical clearance was deemed unnecessary by the University of Zambia Biomedical Research Ethics Committee.

## Results

### Summary of influenza surveillance results during the evaluation period

From January 2011 to December 2017, 10,958 patients were enrolled in the ISSS. Of these, 5111 (46.6%) and 5847 (53.4%) had ILI and SARI, respectively. Influenza results were available for 10,212 of 10,958 (93.2%) enrolled patients; 4776/5111 (93.5%) and 5436/5847 (93.0%) among patients with ILI and SARI, respectively. Influenza viruses were detected in 894/10,212 (8.7%) specimens; in 601/4776 (12.6%) and 293/5436 (5.4%) specimens among patients with ILI and SARI, respectively. Of the 894 influenza-positive specimens, 86 (9.6%) were influenza A(H1N1)pdm09, 342 (38.2%) were influenza A(H3N2), 6 (0.7%) were influenza A not-subtyped and 459 (51.3%) were influenza B viruses. The weekly number of influenza-positive specimens is provided in Fig. 1, panel B.

### Questionnaire survey

The questionnaire was completed by 43/44 (97.7%) surveillance staff at sentinel sites. Of these, 35 (81.4%) were nurses, 5 (11.6%) were clinical officers, 2 (4.7%) were paramedics and 1 (2.3%) was a laboratory technician.

### Evaluation of the surveillance system

The overall mean score for the ISSS in Zambia was 2.6 (moderate to good performance) (Table 1).

#### Data quality and completeness

During the evaluation period, of the seven data indicators, six had good performance and one had moderate performance (Table 2). Approximately 70% of identified SARI cases were enrolled in the surveillance program, while the enrollment target was 100%. The enrollment target for ILI cases was exceeded (140% of the target of 5 cases per week and per facility). More than 90% of CIF were accurately completed for key variables (including site, age/date of birth, sex, date of consultation/admission, date of symptoms onset, date of sample collection and signs and symptoms included in the case definitions) and more than 90% of samples collected had available influenza results and a positive ribonucleic protein result (an indicator of presence of human mucosal cells in the sample). The proportion of collected variables included in the WHO minimum data collection standard was 82.3%. Information on the use of antivirals and the presence of some underlying medical conditions were not included in the patients’ CIFs. The mean score for data quality and completeness was 2.9 (moderate to good performance) (Table 1).

#### Timeliness

During the evaluation period, one timeliness indicator had good performance and one had moderate performance (Table 2). Approximately 25% of samples were not received or tested within the expected time period. 86.3% of the samples collected in Lusaka Province were received within the expected time period, whereas only 67.3% of the samples collected in the Copperbelt Province met the expected timeline. No significant difference in the proportion of samples positive for influenza was observed between the sites situated in Lusaka (9.0% 650/7207) and Ndola (8.1%; 244/3005) (*p* = 0.1429). The mean score for data timeliness was 2.5 (moderate to good performance) (Table 1).

#### Representativeness

During the evaluation period, one indicator had good performance and one had weak performance (Table 2). Age representativeness was strong, with patients enrolled across all age groups. Geographic representativeness was poor, with sentinel sites situated only in 2/10 (20.0%) provinces. The mean score for representativeness was 2.0 (moderate performance) (Table 1).

#### Flexibility

During the evaluation period, both indicators had good performance (Table 2). The ISSS was used as a platform to monitor the circulation of respiratory viruses other than influenza (even though only for two years). The mean score for flexibility was 3.0 (good performance) (Table 1).

#### Simplicity

During the evaluation period, of the seven indicators, six had good performance and one (time of enrollment of SARI/ILI cases) had moderate performance (Table 3). The six indicators used to assess the perception of surveillance personnel at sentinel sites to implement different surveillance activities were rated well by responding personnel. Over 31% of staff, however, reported that surveillance procedures from the identification of cases to the final packaging of samples took > 30 min, showing only moderate performance. The mean score for simplicity was 2.8 (moderate to good performance) (Table 1).

#### Acceptability

During the evaluation period, all four evaluated indicators had good performance (Table 3). More than 90% of surveillance staff was satisfied or very satisfied with the surveillance reports or the feedback of laboratory results. Surveillance activities did not pose a heavy workload on surveillance staff in addition to the required clinical work. The mean score for acceptability was 3.0 (good performance) (Table 1).

#### Stability

During the evaluation period, of the eight indicators, five had good performance and three had moderate performance (Table 4). All surveillance sites provided samples weekly during the evaluation period. The main aspects that affected stability were elevated frequencies of electricity cuts and generator failures. Approximately 30% of surveillance staff did not receive annual refresher training during the last year and approximately 30% of expected weekly reports were not sent to the Zambia MoH within the expected time frame (one week from the end of the reported period). The mean score for stability was 2.6 (moderate to good performance) (Table 1).

#### Utility

During the evaluation period, of the four indicators, three had good performance and one had moderate performance (Table 5). In terms of international utility the Zambia ISSS reported regularly to the WHO FluNet, generated samples that were shared with the WHO collaborating centers and contributed to regional studies. In terms of domestic utility, 4/5 (80.0%) key national objectives of the ISSS were met at the time of this evaluation. The mean score for utility was 2.7 (moderate to good performance) (Table 1).

#### Sustainability

During the evaluation period, the two indicators had poor performance (Table 5). Only 17% of the ISSS budget was provided by the Zambia MoH and the remaining by international agencies. The mean annual running cost of the system was approximately $310,000 ($105 per sample collected and tested in 2017). Whereas, a sustainability plan was drafted in 2015, the document was not finalized and implemented at the time of this evaluation. The mean score for sustainability was 1.0 (weak performance) (Table 1).

## Discussion

We evaluated the performance of the Zambia ISSS over a 7-year period, 3 years from its inception. Overall, based on the evaluated indicators, the system performed satisfactorily with a mean system score of 2.6 (moderate to good performance). The system demonstrated its utility according to its objectives by (i) monitoring the temporal trends of influenza virus circulation; (ii) monitoring the circulating influenza virus types and subtypes annually and enabling the detection of pandemic influenza A(H1N1)pdm09 in 2009 [3] and in subsequent years, (iii) assessing the contribution of seasonal influenza viruses to mild (ILI) and severe (SARI) respiratory illness; (iv) assessing the burden of influenza-associated illness [14]; and (v) generating isolates to contribute to the annual influenza vaccine formulation. Nonetheless, the ISSS was not able to identify groups at increased risk of influenza-associated severe illness (as the necessary variables were not collected in the CIF), one of the objectives of the surveillance system. Strengths of the system included the quality of data generated, its flexibility to monitor viral pathogens other than influenza viruses [24], its potential expandability, its stability over the review period, and its relatively low cost. Identified weaknesses were poor geographic representativeness, lack of timeliness in shipping samples from remote sites, and low sustainability in the absence of external funds.

While quality and completeness of collected key epidemiological and virological data was good overall, an under-enrollment of SARI cases was noted. Many factors could have affected site performance including low staff numbers, high turnovers rates of staff as well as participant refusals. It should be noted that nurses employed by the MoH and tasked with surveillance activities also provided direct patient care. Having dedicated surveillance staff may increase enrollment rates. However, hiring new staff would decrease the sustainability of the surveillance system.

The surveillance system performed moderately in terms of timeliness in transporting samples from the field to the laboratory. Timeliness was mainly affected by the delays experienced from sites located outside Lusaka. Conversely, when samples were received at the central laboratory the processing time was overall satisfactory. While timeliness of shipping of samples from sites situated outside Lusaka Province could be improved by using couriers on a more regular basis this may increase costs. Consideration should be given to timeliness of the system and its geographical representativeness especially in the African setting where more regular shipments of samples from areas distant from central locations are not only affected by cost, but also by logistical constraints.

The geographic representativeness of the system was weak. Establishing additional surveillance sites in other key areas of the country could provide a better representativeness of the circulating influenza viruses. However, it is unclear whether expanding the number of surveillance sites would improve the virological and epidemiological understanding of influenza in Zambia. In a study that included surveillance data from 15 African countries, the detection rate of influenza virus among SARI and ILI cases was similar across the participating countries, suggesting that similarities of the burden of influenza-associated illness across different geographical areas of the continent may be expected [15]. Increasing the number of surveillance sites in Zambia should be considered in light of increased cost, logistical constraints (e.g. regular supervision and transportation of specimens) and impact on laboratory testing capacity and potentially on data quality. Instead of expanding the geographic coverage of the system, efforts could be targeted to improve timeliness of sample shipment and enrollment of patients meeting the SARI case definition at the existing sentinel sites as well as collecting more detailed epidemiological information on enrolled cases (e.g., relevant risk factors for influenza-associated severe illness and in-hospital outcomes that are recommended by the WHO guidelines for global influenza surveillance, but are currently not fully captured by the system). In particular, the collection of additional information on underlying medical conditions may enable the continuous monitoring of high risk groups for influenza-associated severe illness. This would also align the Zambia ISSS to the minimum data collection requirements suggested by WHO [6].

The flexibility of the system was demonstrated by its ability to investigate the viral etiology of pathogens other than influenza [24]. While the recruitment of dedicated surveillance officers may not be justifiable for a vertical influenza surveillance program it may be considered for an integrated multi-pathogen surveillance platform. This could be potentially cost-beneficial for a sentinel surveillance system where a limited number of surveillance officers may be needed in selected sentinel sites as compared to national passive surveillance programs. Integrated and well-run multi-pathogen surveillance systems, if adequately staffed may provide high quality and timely epidemiological and virological data with a “modest” financial investment. The national burden of illness attributable to specific pathogens can be estimated from sentinel surveillance data through special studies as demonstrated in Zambia and other African countries for influenza [10–16]. This would be particularly relevant for policy makers if multiple pathogens are included so as to provide the relative burden associated with different etiological agents. Risk factors for influenza-associated severe illness (e.g. hospitalization or death) have also been assessed using sentinel surveillance systems [23, 25–27] and this could be implemented also in Zambia for influenza and other pathogens.

The stability of the system was demonstrated by its ability to enroll and process samples every week of the review period. This may be related to the simplicity and acceptability of the system and the availability and use of standard operating procedures at sites. Nonetheless, tasking of surveillance staff with clinical and surveillance work may have affected the enrollment rate as previously discussed.

## Conclusion

In conclusion, the system performed satisfactorily over recent years. It contributed to the body of knowledge on the burden of influenza [3, 14] and other respiratory viruses [24] among common respiratory syndromes in Zambia and globally through sharing of influenza data [8, 28] and virus isolates. Nonetheless, despite its moderate cost, the ISSS in Zambia is largely reliant on external funds and the acceptability of maintaining the surveillance system through national funds would require evaluation. Improvement of the system would entail the improvement of the timeliness of sample shipments, enrollment of patients meeting the SARI case definition and an increased geographical coverage; however, the latter would increase costs and impose logistical constraints. Furthermore, while the surveillance system is stable and able to identify circulating influenza strains, the data being generated is not fully utilized as Zambia lacks guidelines on antivirals use and vaccination policy for influenza. A better use of the available data, in association with special studies, could be made to inform and promote mitigation interventions.

## Additional file

**Additional file 1.** QUESTIONNAIRE FOR THE SITE PROVIDER(S)

## Data Availability

All data generated or analyzed during this study are included in this published article.

## Abbreviations

CDC: Centers for Disease Control and Prevention
CIF: Case Investigation Form
HA: Haemagglutination
HAI: Haemagglutination Inibition
hMPV: Human Metapneumovirus
ILI: Influenza-Like Illness
ISSS: Influenza Sentinel Surveillance System
MoH: Ministry of Health
MS: Microsoft
NIC: National Influenza Center
PIV: Parainfluenza Virus
RSV: Respiratory Syncytial Virus
RT-PCR: Reverse Transcription Polymerase Chain Reaction
SARI: Severe Acute Respiratory Illness
WHO: World Health Organization
