# Supplementary material for: An evaluation of the Zambia influenza sentinel surveillance system, 2011–2017
Source: BMC Health Serv Res. 2020 Jan 13;20:35. doi: 10.1186/s12913-019-4884-5 (PMC6958603; doi:10.1186/s12913-019-4884-5)
Supplement: Supplementary file 1 — Additional file 1. QUESTIONNAIRE FOR THE SITE PROVIDER(S) [file 12913_2019_4884_MOESM1_ESM.docx]

**QUESTIONNAIRE FOR THE SITE PROVIDER(S)**

*Evaluation of Zambia Influenza Sentinel Surveillance System, 2015*

| **A.1 Date of the survey:** /__/__/__/__/2/0/1/5/  **A.2 Site reference:** /…………../  **A.3 Name of the center:** /____________________________/  **A.4 District:** /____________________________/  **A.5 Province:** /____________________________/  **A.6 Site Type:** clinic 🞏 Hospital 🞏  A.7 Type surveillance that you implement (Tick all that apply)  SARI 🞏 ILI 🞏 both 🞏  **A.8 What type of surveillance is implemented at your influenza site?** ILI 🞏 SARI 🞏 BOTH  **A.10 staff category (one or more answers possible):**  Medical officer 🞏 Nurse 🞏 Clinical officer 🞏 Laboratorian 🞏  Other (specify):………………………………………. | /__ /  /__ /  /__ /  /__ /  /__ /  /__ /  /__ /  /__ /  /__ /  /__ / |
| --- | --- |

| **B.1 Have you undergone training in the implementation of sentinel surveillance activities?**  yes 🞏 no 🞏  **If “yes”, please specify the last year of training:** less than 1 year ago 🞏 less than 2 years ago 🞏  More than 2 years ago | /__ /  /__ /  /__ /  /__ /  /__ /  /__ / |
| --- | --- |

| **C.1 How long have been working in influenza surveillance?**  Less 6months 🞏 1 year 🞏 1-2years 🞏 more than 2 years 🞏  **C.2 Do you experience difficulties completing the individual CIF forms?**  yes 🞏 no 🞏  **If “yes”, what are the main reasons preventing the completion of CIF forms?**  Absence of personnel 🞏 Workload 🞏  New person in charge 🞏 Logistical problems 🞏  Other (specify): ……………………………………………………… | /__ /  /__ /  /__ /  /__ / |
| --- | --- |

| **D.1 How do you find:**  **a) Identification of cases**  Difficult 🞏 Somewhat difficult 🞏 Somewhat easy 🞏 Easy 🞏  **b) Consenting of patients**  Difficult 🞏 Somewhat difficult 🞏 Somewhat easy 🞏 Easy 🞏  **c) Completion of forms (individual CIF forms)**  Difficult 🞏 Somewhat difficult 🞏 Somewhat easy 🞏 Easy 🞏  **d) Collection of samples**  Difficult 🞏 Somewhat difficult 🞏 Somewhat easy 🞏 Easy 🞏  **d) Sample packaging**  Difficult 🞏 Somewhat difficult 🞏 Somewhat easy 🞏 Easy 🞏  **e) Sending of samples**  Difficult 🞏 Somewhat difficult 🞏 Somewhat easy 🞏 Easy 🞏  **If the response what “difficult” or “somewhat difficult,” please explain why.**  ………………………………………………………………………………………………………………………………….………………………..  **D.2 Did the transportation service comply with the scheduled pick-up of boxes/samples?**  Always 🞏 Often 🞏 Rarely 🞏 Never 🞏  ………………………………………………………………………………………………………………………………….………………………..  …………………………………………………………………………………………………………………………………..………………………..  **D4 How long does it take you to complete :**  **a) Identification of cases**  less than 30min 🞏 30min-1 hour 🞏 more than 1 hour 🞏 2 hours and above 🞏  **b) Completion of forms (individual CIF forms)**  less than 30min 🞏 30min-1 hour 🞏 more than 1 hour 🞏 2 hours and above 🞏  **c) Sample collection and packaging**  less than 30min 🞏 30min-1 hour 🞏 more than 1 hour 🞏 2 hours and above 🞏  **D5 How many SARI cases do you enroll per day(average);**  Up to 5 cases 🞏 6-10 cases 🞏 11-15 cases 🞏 over 15 cases 🞏  **D6 How many ILI cases do you enroll per day(average);**  Up to 5 cases 🞏 6-10 cases 🞏 11-15 cases 🞏 over 15 cases 🞏 | /__ /  /__ /  /__ /  /__ /  /__ /  /__ /  /__ /  /__ /  /__ /  /__ / |
| --- | --- |

| **E.1 Did you receive information/feedback regarding the following:**  **a) Influenza test results:**  Regularly 🞏 Sometimes 🞏 Rarely 🞏 Not at all 🞏  **b) Influenza bulletin**  Regularly 🞏 Sometimes 🞏 Rarely 🞏 Not at all 🞏  **c) Epicurve/graph/table**  Regularly 🞏 Sometimes 🞏 Rarely 🞏 Not at all 🞏  **How did you utilize feedback/information given?**  ………………………………………………………………………………………………………………………………….………………………..  …………………………………………………………………………………………………………………………………..………………………..  ………………………………………………………………………………………………………………………………….………………………..  …………………………………………………………………………………………………………………………………..……………………….. | /__ /  /__ /  /__ /  /__ /  /__ / |
| --- | --- |

| **F.1 Are you satisfied with the feedback provided regarding the following?:**  **a) Reception of influenza test results:**  Satisfied 🞏 Somewhat satisfied 🞏 Mostly unsatisfied 🞏 Not at all satisfied 🞏  **b) Influenza bulletin**  Satisfied 🞏 Somewhat satisfied 🞏 Mostly unsatisfied 🞏 Not at all satisfied 🞏  **- Overall satisfaction scale** (out of 10)**: 1/2/3/4/5/6/7/8/9/10/***(circle the response)*  **- If the response is «not satisfied» or «mostly not satisfied», please explain why?**  *(*  ………………………………………………………………………………………………………………………………………………………………..  ………………………………………………………………………………………………………………………………………………………………..  **F.2 With regards to your weekly activities, how much time do you dedicate to sentinel surveillance activities?  ________ hour(s) per week or percentage ____________%** | /__ /  /__ /  /__ /  /__ /  /__ /  /__ / |
| --- | --- |

| **G.1 Does your center have the following?**  **Influenza sampling procedure**: yes 🞏 no 🞏  **Decision tree:** yes 🞏 no 🞏  **G.2 Do you use standardized tools for influenza surveillance?**  **a) Influenza sampling, storage, and transportation procedures**  yes 🞏 no 🞏  **c) Decision tree:**  yes 🞏 no 🞏  **If the response is “no,” please explain why.**  ………………………………………………………………………………………………………………………………….  …………………………………………………………………………………………………………………………………..  **G.3 Did your site ever experience a shortage in the following supplies?**  **a) Forms:**  Never 🞏 Once per year 🞏 2-3 times per year 🞏 More than 4 times per year 🞏  **b) Influenza sampling kits:**  Never 🞏 Once per year 🞏 2-3 times per year 🞏 More than 4 times per year 🞏  **c) power supply:**  once a week 🞏 2-3 times a week 🞏 more than 3 times a week 🞏 never 🞏**d) Others:…………………………………………………………(please specify)** | /__ /  /__ /  /__ /  /__ /  /__ /  /__ /  /__ /  /__ /  /__ /  /__ /  /__ / |
| --- | --- |

| **IN GENERAL, WHAT ARE YOUR OVERALL IMPRESSIONS OF THE SENTINEL SURVEILLANCE SYSTEM?** | /__ / |
| --- | --- |
| ………………………………………………………………………………………………………………………………………………………………  ……………………………………………………………………………………………………………………………………………………………….  ………………………………………………………………………………………………………………………………….…………………………….  ………………………………………………………………………………………………………………………………….…………………………….  ………………………………………………………………………………………………………………………………….…………………………….  ………………………………………………………………………………………………………………………………….……………………………. |  |

| **DO YOU HAVE ANY SUGGESTIONS FOR IMPROVING THE SURVEILLANCE NETWORK’S OPERATION?** | /__ / |
| --- | --- |
| ………………………………………………………………………………………………………………………………….…………………………….  ………………………………………………………………………………………………………………………………….…………………………….  ………………………………………………………………………………………………………………………………….…………………………….  ………………………………………………………………………………………………………………………………….…………………………….  ………………………………………………………………………………………………………………………………….…………………………….  ………………………………………………………………………………………………………………………………….…………………………….  ………………………………………………………………………………………………………………………………….……………………………. |  |

***Thank you for your participation!***
